# Thermodynamic and kinetic study of adsorptive removal of lead by the nanocomposite loaded nanofibers

**DOI:** 10.3906/kim-2107-67

**Published:** 2021-11-10

**Authors:** Urwa MAHMOOD, Sharjeel ABID, Bilal QADIR, Ahsan NAZIR, Tanveer HUSSAIN

**Affiliations:** Electrospun Materials & Polymeric Membranes Research Group, National Textile University, Faisalabad, Pakistan

**Keywords:** Electrospun nanofibers, nanocomposites, heavy metals, adsorption, bentonite, fly ash

## Abstract

Herein, the fabrication of electrospun nanocomposites, using polyacrylonitrile nanofibers (PNF) modified with nano-bentonite and fly ash, is explained. Further, the use of electrospun adsorbent for the remediation of Pb (II) ions from water has been explored. Pristine PNF and nanocomposites were characterized using SEM, EDX, and FTIR to analyze surface topology, elemental composition, and functional groups, respectively. The adsorptive behavior of developed adsorbents was investigated using the effects of dosage, initial concentration, time, and temperature. Pseudo-second order kinetics fit well with experimental data and the adsorption followed intra-particle diffusion. The thermodynamics study confirmed spontaneous endothermic adsorption of the heavy metal. Nanocomposites-based adsorbents showed improved adsorption capacity for Pb (II) ions compared to pristine PNF.

## 1. Introduction

Hazardous discharge from industries is the primary cause of heavy metal contamination in water, which has gained considerable importance in the last decades. These contaminations are caused by various industrial activities. These activities are the major contributors of heavy metals contamination, including lead, chromium, arsenic, zinc, mercury, cobalt, copper, and others [1]. The effluents from these industries contain a very high concentration of harmful heavy metals, much higher than the limits defined by the World Health Organization (WHO) [2].

Heavy metals are toxic and, in many cases, are carcinogenic towards living beings. Lead, one of the most commonly found toxic metals, has hazardous effects on living beings even at low concentrations [3]. About 5%–15% of lead taken up by humans enters the body through the digestive route, while 20%–80% enters through the respiratory tract. Food containing lead ions causes absorption of lead within the body, especially lungs and stomach, enters into the bloodstream, adheres to blood cells, and forms clots in joints and bones. Their intervention with different body organs results in anemia, kidney disorder, liver damage, adverse effects on the reproductive system, mental illness, and in severe cases, cancer in adults. Woefully, it also causes various treacherous effects on children, including abnormal brain development, brain swelling, severe disabilities, dental issues, abnormal behavioral changes, and encephalopathy [4]. These facts suggest the development of water treatment systems to remove lead from wastewater.

Different techniques have been devised for the treatment of heavy metals, including lead from wastewater. These includes ion exchange [5], chemical precipitation [6], coagulation [7], flocculation [8], membrane separation [9], ultra-filtration, and oxidation-reduction process [10,11]. However, these techniques offer low removal efficiency, higher operation, and maintenance costs, result in a larger amount of waste, and pollute the ecosystem. Further, they may require secondary treatments in addition to the primary treatments making the process highly expensive. Different approaches have been proposed to overcome these problems and are more efficient and cheaper [12]. Most of these techniques are based on the adsorption process and offer advantages, including cost-effectiveness, flexibility, and high purity of the treated water [13, 14]. The advantages of the adsorption process can be multiplied using nanomaterials thanks to their exceptional properties, including high surface-to-area ratio, tunable porosity, easy fabrication, and wide range of choices for the surface functionalization [15].

Several researchers explored the potential of different nanomaterials for the lead adsorption. Lai et al. reported a biomass composite having a porous structure incorporated with titanium oxide functionalized graphene oxide for the decontamination of lead in wastewater and showed good absorption (132 mg.g^−1^). The composite was regeneratable at mild conditions and good thermal stability and had high lead uptake even after several repetition cycles. At the same time, the adsorption was followed by the pseudo-second-order kinetics and Langmuir model [16]. Gebru et al. fabricated cellulose acetate (CA) nanofibers incorporated with titanium oxide (TiO_2_) for the adsorption of Pb (II) and Cu (II). As a result of increasing the TiO_2_ above 2.5%, adsorption efficiency reduces. The reduction in adsorption efficiency was due to the agglomeration, which decreases porosity and surface area [17]. Li et al. developed electrospun PAN nanofibers loaded with MnO_2_ using polydopamine coating to capture Pb (II) ions. The adsorption capacity of the adsorbent was 185 mg.g^−1^ as calculated by Langmuir isotherm [18].

Electrospun nanomaterials t can immobilize heavy metal impurities, including lead using adsorption process [19, 20]. Especially, the capability of electrospun materials to be functionalized allows them to act as adsorbent materials for the immobilization of lead ions. Additionally, these materials offer several advantages, including low manufacturing and operating costs, effortless operation, low energy consumption, ease of maintenance, and lower carbon footprint [21]. Numerous approaches have been used to improve the properties of electrospun nanomaterials compared to conventional materials. These include modifications, a combination of polymers or additives, surface coatings, functionalized additives, and polymers [22]. Especially the electrospun nanomaterials developed using polymeric adsorbents modified with different additives have drawn considerable attention for wastewater treatment. For example, researchers have fabricated composite nanofibers containing polycaprolactone impregnated with clay and zeolite having a synergistic effect on the adsorption capacity of Pb (II). The results showed that adsorption was a spontaneous process and observed the Freundlich model and pseudo-second order kinetics [23]. Similarly, Thamer et al. improved the adsorption of Pb (II) using carbon nanofibers (CNFs) functionalized with poly(m-phenylene diamine) and melamine. The poly(m-phenylene diamine) based materials showed spontaneous and endothermic adsorption while the melamine functionalized sample materials exothermic adsorption [24].

Researchers have used different polymers to develop electrospun nanomaterials for heavy metal adsorption from wastewater, for example, polyamide 6 (PA6), polyvinylidene fluoride (PVDF), and others. Among these materials, polyacrylonitrile (PAN) is considered one of the most suitable candidates for electrospinning thanks to its low cost and exceptional chemical, thermal and mechanical stability; however, its adsorption properties can be improved further towards heavy metals [25]. To improve the Pb (II) adsorption of electrospun PNF, the current study employed a composite of Smectites (bentonite) and fly ash that was expected to work synergistically to enhance lead adsorption. Bentonite possesses a higher cation exchangeability for heavy metals and is readily available at a low cost [12]. Similarly, fly ash, a waste material of various industrial activities, is known to provide adsorption sites to remove Pb (II) [26]. A nanocomposite consisting of PAN, bentonite, and fly ash was produced and cross-linked to improve the adsorption properties. The developed nanocomposites were evaluated for lead adsorption at different doses, contact time, and temperatures. Further, adsorption kinetics, isotherms, and thermodynamics parameters of the developed nanocomposites were studied.

## 2. Experimentation details

### 2.1. Materials

Polyacrylonitrile (PAN, analytical grade, molecular weight (MW) 150,000 g/mol and density (ρ) 1.184 kg/cm^3^) was procured from Exlan Corp, Japan, while dimethylformamide (DMF, GR grade, purity 99.5% and ρ 0.0944 kg/cm^3^) were obtained from Sigma Aldrich Germany. Bentonite Clay was purchased from DAEJUNG chemicals, Korea, and fly ash was collected from Sitara Chemicals, Pakistan. Lead Nitrate (purity 99.0% & MW 331.21) were obtained from Sigma Aldrich Germany. Epichlorohydrin (GR grade, purity 99.0%) was procured from DUKSAN reagents, Korea.

### 2.2. Fabrication of mixed matrix membranes

#### 2.2.1. Development of nanocomposites

Bentonite and fly ash were blended with different bentonite to fly ash ratios of 80:20, 40:60, 60:40, and 20:80 using a mortar piston. The blended samples were then calcinated in a quartz tube furnace (PT-1200T, Zhengzhou Protech Technology Co., Ltd. China) at a ramp of 10 °C/min up to 700 °C and kept at 700 °C for 300 min.

#### 2.2.2. Fabrication of PAN nanofibers

**Figure 1:**
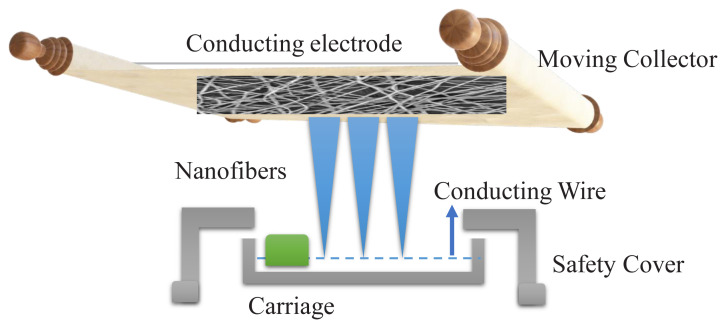
Needleless electrospinning process.

To prepare 8% (wt/v) PAN dope solution, the required amount of PAN precursor was dissolved in 60 mL of DMF followed by vigorous stirring for 12 h. The homogenized solution was then subjected to Nanospider electrospinning (ELMARCO, Czech Republic) with varying parameters. Nanofibers were optimized to a minimum diameter which reduces the fiber-to-fiber diameter and increases the contact time of adsorbents with Pb (II) ions. After the preliminary trials, the minimum diameter of nanofibers was achieved at the following parameters carriage speed of 80 mm/s. The distance between the spinning electrode and substrate was 200 mm with 30 kV voltage. A schematic diagram of electrospinning is shown below in Figure 1. Electrospun webs were collected on an aluminum file and placed in an oven at 60 °C for 5 h to evaporate the solvent and then peeled off from an aluminum file. All the electrospun webs were stored in an airtight container to prevent external contamination and future use.

#### 2.2.3. Crosslinking of PAN

The pristine PAN nanofibers were treated with 2.5% epichlorohydrin (ECH) solution to cross-link and coated with bentonite fly ash nanocomposites then subjected to drying in an oven (Thermo Fisher Scientific, United States) at 60 °C. The developed adsorbents with compositions 80:20, 40:60, 60:40, and 20:80 are NC1, NC2, NC3, and NC4, respectively.

### 2.3. Material characterizations

#### 2.3.1. Scanning electron microscopy (SEM)

Adsorbents were placed on stubs using conductive adhesive tape and gold-coated for 30 s using a sputter coater (Desk V). SEM (Nova, nanoSEM 450, FEI Czech Republic) with secondary electron detector mode was used at 10 kV for the fabricated adsorbents’ morphological and surface textural analysis.

#### 2.3.2. Energy dispersive x-ray spectroscopy (EDX)

For the elemental analysis of developed adsorbents, EDX (Oxford INCA X’Act) was used.

#### 2.3.3. Fourier transform infrared spectroscopy (FTIR)

Pristine PAN nanofibers and nano impregnated adsorbents were analyzed on FTIR (ZnSe-HATR Module, Perkin Elmer-Spectrum two, USA) with an average of 20 scans in the scanning range of 4000-600 cm^−1^ and resolution of 4 cm^−1^ for the identification of chemical structure.

#### 2.3.4. Batch adsorption studies

Batch adsorption studies was used to analyze the adsorption capacity of developed adsorbents. A stock solution of Pb (II) of 100 ppm was prepared using 1% nitric acid and lead nitrate salt to optimize the dosage of adsorbents at 20 °C temperature for 8 h. Further, the effect of time and temperature on the adsorption was studied. After the adsorption, adsorbate was filtered and diluted prior to ICP analysis (ICP-OES 5110, Agilent, USA). The adsorption kinetics was performed under the optimized conditions while the thermodynamics of the adsorption process were investigated from 20 °C to 45 °C. After adsorption experimentations, lead concentrations were determined using ICP analysis at a wavelength of 220.353 nm [27]. Calibration solutions of 0.25 ppm, 0.5 ppm, 1 ppm, 1.5 ppm, and 2 ppm were prepared using 1% nitric acid and lead nitrate salt.

The adsorbed amount of Pb (II) and adsorption efficiency (%) of adsorbents were calculated using the following relations [28].


Equation 1 qt=(C0-Ce)VM

Equation 2 Removal efficiency (%)=(C0-Ce)C0×100

Where *V* is the volume of solution in liters (L), *M* is the weight of adsorbent in grams (g), *q_t_* is the adsorbate amount adsorbed per unit time, *C_0_* and *C_e_* are the initial and equilibrium concentrations in mg per g (mg.g^−1^), respectively.

#### 2.3.5. Thermodynamics

Thermodynamics analysis provides the information about the adsorption behavior and the parameters of thermodynamics include Activation entropy (*ΔS’*), enthalpy (*ΔH’*), and Gibbs free energy (ΔG’) and can be calculated in following relations [29].


Equation 3 Ke=qeCeq

Equation 4 $$\Delta G^{\prime }=-R.T.lnK_{e}$$

Equation 5 $$\Delta G^{\prime }=\Delta H^{\prime }-T.\Delta S^{\prime }$$

Equation 6 ln Ke=ΔS′R-ΔH′RT

Here, *q_e_* (mg.g^−1^) is the adsorbed concentration at equilibrium, *C_eq_* (mg.g^−1^) is the equilibrium concentration, *R* (K.J.mol^−1^.K^−1^) is considered as ideal gas constant, T (K) and *K_e_* are absolute temperature and equilibrium constant, respectively. The ΔG’ and ΔH’ values can be estimated by plotting a linear curve of Van’t Haff plot (ln *K_e_* vs 1/T) [30]. However, the equilibrium constant (*K_e_*) can be calculated using the Equation 3.

#### 2.3.6. Adsorption kinetics

Kinetic models have been proposed to determine the mechanism of the adsorption process that provides valuable data such as reaction pathways to improve the efficiency and feasibility for the scale-up of the adsorption process [31,33]. To serve this purpose, five different kinetic models, i.e. pseudo-first order model, pseudo-second order model, Elovich model, Bangham’s model, and intra-particle diffusion model, were employed on the experimental equilibrium adsorption data. All the models were applied in their linearized forms.

The pseudo-first order model was employed on the dynamic data to estimate the rate of Pb (II) adsorption rate on pristine PNF, NC1, NC2, NC3, and NC4. This model provides key information about the diffusion of adsorbate through the adsorbent interface. The linear form of the pseudo-first order kinetic model is given in Equation 7 [34].


Equation 7 $$ln(q_{e}-q_{t})=lnq_{e}-k_{1}t$$

whereas, *q_e_* (mg.g^−1^), *q_t_* (mg.g^−1^) are the adsorption capacity at equilibrium and at a time (t), *k_1_* (min^−1^) is the pseudo-first order constant and can be estimated by plotting t vs. *ln(q_e_ - q_t_*). To understand the kinetics of Pb (II) adsorption on pristine and NF composite, pseudo-second order model was applied linearly on equilibrium adsorption data using the following expression [35].


Equation 8 $$\frac{t}{q_{t}}=\frac{1}{k_{1}q_{e}^{2}}+\frac{t}{q_{e}}$$

Where *q_t_* (mg.g^−1^) and *q_e_* (mg.g^−1^) are the instantaneous and equilibrium adsorption capacity of nanocomposites and *k_1_* (g.mg^−1^. min) is the constant associated with the pseudo-second order model.

Bangham model was applied on the equilibrium adsorption data to evaluate adsorption mechanisms such as pore diffusion. The linear form of the Bangham’s model is represented as follow [36]:


Equation 9 $$\text{log }\left(\frac{C_{i}}{C_{i}-q_{t}M}\right)=\text{log }\left(\frac{k_{1}}{2.303V}\right)+\beta logt$$

Where *C_i_* (mg.L^−1^) is the initial concentration of Pb (II) ions in the solution, and M (g) is the mass of adsorbent, V (L) is the volume of solution whereas, *β* is the constant of the Bingham model. Similarly, intra-particle diffusion (Weber and Morris) was employed to distinguish various diffusion mechanisms to identify the dominant rate-limiting step for Pb (II) adsorption on new PNF, NC1, NC2, NC3, and NC4. The intra-particle diffusion model can be represented as [36].


Equation 9 $$q_{t}=k_{1}\sqrt{t}+B_{1}$$

Where, *k_1_* (mg.g^−1^.min^½^) the constant of the model and *B_1_* (mg.g^−1^) is the thickness of the boundary layer. The large value of *B_1_* implies that the boundary layer has a prominent influence on the Pb (II) ion adsorption.

## 3. Results and discussion

### 3.1. FTIR

**Figure 2:**
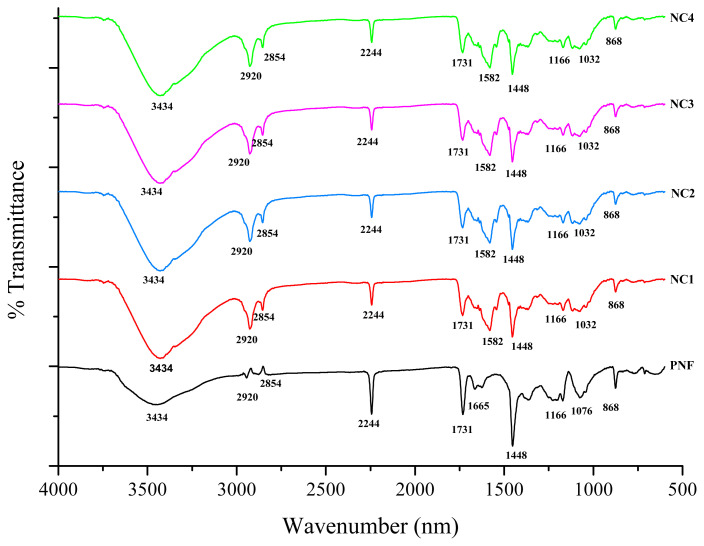
FTIR spectra of developed samples.

FTIR spectra of pristine PNF, NC1, NC2, NC3, and NC4 are shown in Figure 2. In the FTIR spectra of pristine PNF, the broad transmittance peak at 3434 cm^−1^ indicates the −OH stretching due to absorbed moisture from the atmosphere. The transmittance peak at 2854 cm^−1^ and 2920 cm^−1^ corresponds to symmetric and asymmetric −C-H stretching [37]. The characteristic peak at 2244 cm^−1^ is ascribed to the vibrational stretching of −C≡N, and sharp peaks at 1731 and 1448 cm^−1^ correspond to the vibrational stretching of −C=O and −C–H_,_ respectively [38,39]. The peak at 1665 cm^−1^ is assigned to −C=C stretching, which disappears in the spectra of cross-linked nanofibers [40]. The peak of strong −CH_2_ stretching is observed at 1076 cm^−1^ [41]. However, after the cross-linking of PNF with ECH, some significant changes have been observed in the FTIR spectra of NC1, NC2, NC3, and NC4. The transmittance peak that appears at 1582 cm^−1^ corresponds to the −CH_2_ vibrations due to the cross-linking of pristine PNF with ECH [42]. Further, the spectrum of NC1, NC2, NC3, and NC4 conforms to the absorption band at 1044 cm^−1^ attributed to stretching vibrations of Si-O, SiO - SiO, and Si-Si groups [43, 44]. The FTIR analysis confirms the successful cross-linking of PNF without modifying the surface functionalities.

### 3.2. Surface topology (SEM analysis)

**Figure 3:**
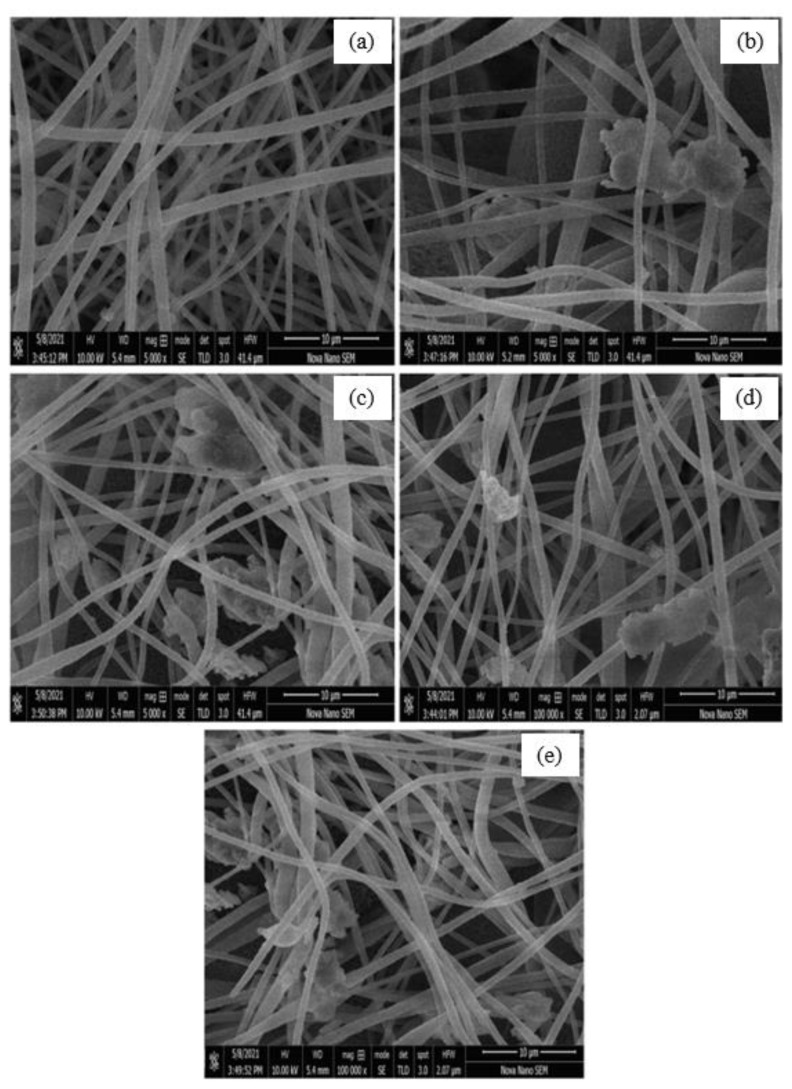
SEM micrographs of (a) pristine PNF, (b) NC1, (c) NC2, (d) NC3, (e) NC4.

The topological highlights of PNF, NC1, NC2, NC3, and NC4 are shown in Figures 3 (a)–3(e), respectively. The micrograph of PNF (Figure 3 a) exhibits the smooth, nonporous surface and bead-free structure of nanofibers. However, after the loading of nanocomposite on nanofibers, agglomeration can be observed in the micrographs of NC1, NC2, NC3, and NC4, as shown in Figures 3 (b)–3 (e), respectively. It was concluded by scanning electron microscopic analysis that there was a successful loading of NC on nanofibers, as can be seen in Figure 3. The diameter of the PNF and NC was analyzed by using Image J (1.53 e) software. The diameter of PNFs and NCs is summarized in Table 1.

### 3.3. Energy dispersive X-ray (EDX)

The EDX analysis of nanocomposite-based adsorbents is summarized in Table 2. In PNF, no traces of any metallic impurities have been observed. However, with the addition of nanocomposite (Flyah-Bentonite), metallic constituents of nanocomposite are observed, as evident from Table 2. The presence of these metallic constituents in NC1, NC2, NC3, and NC4 confirmed the successful incorporation of nanocomposite in the electrospun nanofibers.

### 3.4. Effect of dosage

**Figure 4:**
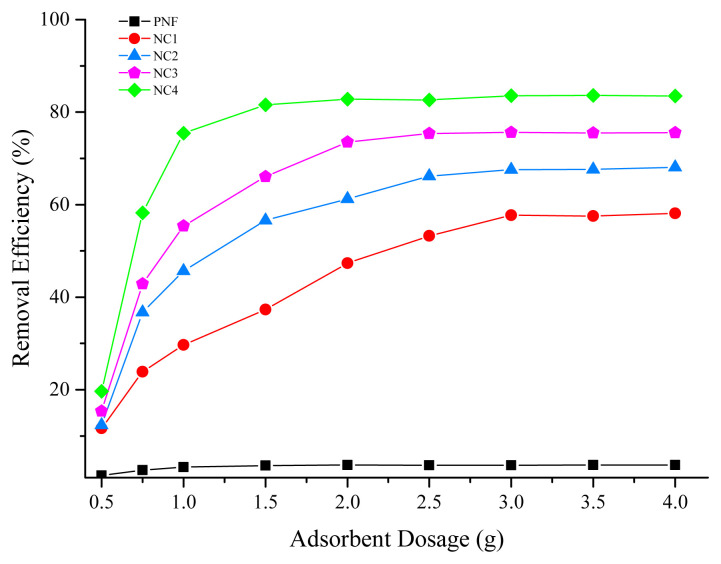
Effect of dosage on the adsorption of Pb (II) ions.

The dosage of adsorbent has a prominent effect on the adsorption capacity of Pb (II) ions and is demonstrated in Figure 4. In the case of PNF, the percentage removal remains constant at about 3% even after increasing the dosage due to the less number of active sites [45,46]. However, the percentage removal of NC1, NC2, NC3, and NC4 increases considerably with the increase in adsorbent dosage owing to the availability of vulnerable sites for adsorption [47]. NC4 showed up to 76% efficiency, at a dosage of 1 g as compared to PNF, NC1, NC2, and NC3, adsorbents owing to the availability of active sites. Further increasing the dosage showed no significant improvement in the Pb (II) adsorption [48,49]. Hence, the dosage of 1 g nanocomposites was selected for further studies as the maximum efficiency was achieved at 1 g after increasing the dosage above 1 g, adsorption becomes stagnant with a little change as observed in the Figure 4.

### 3.5. Adsorption kinetics

**Figure 5:**
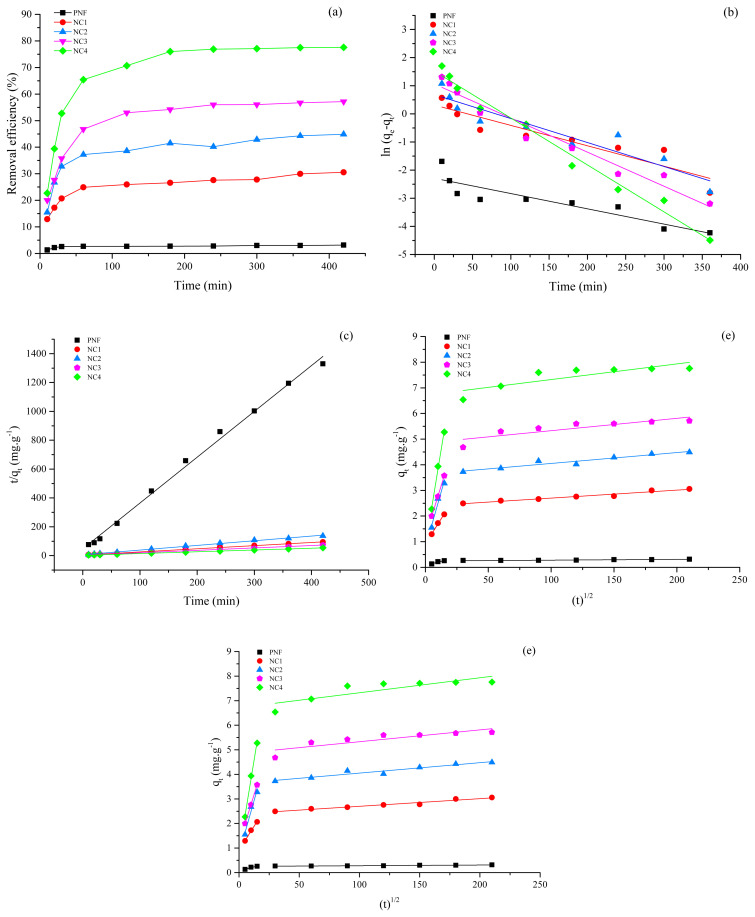
Effect of time and kinetic models’ plots on the adsorption of Pb (II) ions using pristine PNF and different nanocomposites (NC) (a) effect of time up to 420 min at 25 °C with a dose of 1g and initial concentration of 100 ppm at pH 6 (b) pseudo-first order model (c) pseudo-second order model (d) Bangham model (e) intra-particle diffusion model.

The adsorption kinetics were studied by varying the contact time from 10 to 420 min. After each time interval, the adsorbate was filtered and analyzed to measure the adsorption capacity. The equilibrium adsorption time for PNF was 20 min, as shown in Figure 5 (a). No considerable Pb (II) adsorption was observed after the equilibrium time, which may be due to the occupation of all the active sites for the adsorption [49]. However, the equilibrium adsorption time was increased by incorporating the nanocomposite, as shown in Figure 5 (a). Furthermore, the NC4 exhibits the highest equilibrium adsorption time compared to its counterparts which may be ascribed to the highest fly ash concentration in the incorporated nanocomposite, which offers additional susceptible adsorption sites [50]. Addition of nanocomposite increases the available active sites, therefore, more time was required to establish the adsorption equilibrium state [51, 52]. Hence, the adsorption of NC was remarkably enhanced compared to pristine PNF due to bentonite and fly ash composite.

The parametric values of kinetic models have been summarized in Table 3 and the graphical representations are shown in Figures 5(b)–5(e). The modeled data exhibited that the Pseudo-first order model did not comply well with the equilibrium adsorption data, i.e. R^2^ < 0.9 for PNF, NC1, NC2, NC3, and NC4 as shown in Figure 5 (b). The regression coefficient of the pseudo-second order model (R^2^ = 0.99) for PNF, NC1, NC2, NC3, and NC4 depicts a good agreement of modeled data with the equilibrium adsorption data compared to its counterparts, as presented in Figure 5 (c). The compliance of the pseudo-second order model revealed that the rate-limiting step for the adsorption of Pb (II) ions might be the chemisorption which involves electrostatic interactions, including exchangeability of electrons between the Pb (II) ions and adsorbents [53, 54]. Moreover, the Bangham’s model did not find an excellent fitting with the dynamic data, as can be seen in Figure 5 (e), which confirms that the Pb (II) adsorption was not controlled by the pore diffusion mechanism [55]. The plot of the intra-particle diffusion model showed two linear plots, as shown in Figure 5 (f), which demonstrates that there are more than two steps involved in the adsorption of Pb (II). Moreover, the nonzero intercept of the first linear plot suggested that the adsorption process was diffusion based. Furthermore, the first step indicates the rapid diffusion of Pb (II) at external layers. In contrast, the gradual adsorption was noticed in the second step where the interparticle diffusion was the rate-limiting step [56,57]. It shows a complicated mechanism owing to the internal diffusion between particles and mass transfer. The deviation of the line from the origin indicates curves deviate from the origin, which means the adsorption process is not only monitored by the diffusion process. Other factors may also affect the adsorption process [2]. Various studies were reported relating to the discussed kinetic models [58, 59].

### 3.6. Thermodynamics study of adsorption

**Figure 6:**
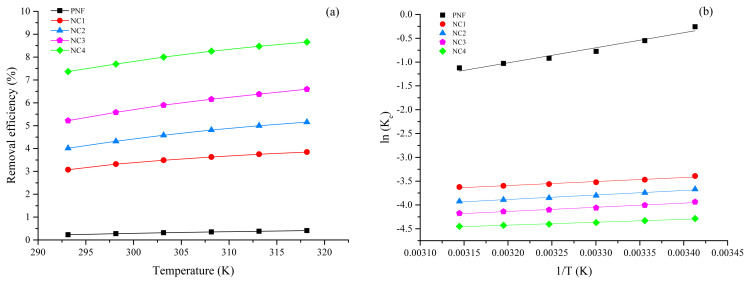
(a) Effect of different temperatures on Pb (II) adsorption (b) Van’t Hoff plot.

The Pb (II) ions adsorption was examined at temperatures, i.e. 20, 25, 30, 35, 40, and 45 °C, as shown in Figure 6 (a). As evident from Figure 6 (a), the removal of Pb (II) increases significantly with the rise in solution temperature. This increase in percentage adsorption is associated with the higher kinetic energy of the Pb (II), which facilitates the adsorption process at higher temperatures [60, 61]. The Pb (II) dehydration is increased at high temperatures, which means the process absorbs heat. Dehydration of Pb (II) ions increases which ultimately facilitates the adsorption [62]. Elevated temperature increases the movement of Pb (II) ions; thus, more kinetic energy is acquired because of the increase in velocity [63].

The parameters of thermodynamics for Pb (II) adsorption are mentioned in Table 4 and the Van’t Hoff plot is represented in Figure 6 (b). The ΔG’ values for pristine PNF over the entire temperature range are positive, which means the adsorption was nonspontaneous. It can be observed that ΔG’ of NC1, NC2, NC3, and NC4 have negative values at all temperatures that indicate the reaction’s spontaneity. However, values of ΔG’ were decreasing as the temperature elevates, which exhibits that the adsorption of Pb (II) is more feasible at higher temperatures owing to the affinity of adsorbents towards Pb (II) [53, 64]. The ΔG’ values for pristine PNF, NC1, NC2, NC3, and NC4 are less than 8.0 kJ/mol, which confirms that the adsorption of Pb (II) on all adsorbents are physical adsorption (physisorption) [65, 66]. In the case of ΔS’, the obtained values were positive, which showed that the process was irreversible, had an affinity towards Pb (II) ions, and increased the degree of freedom at the adsorbent/solution interface [67]. The positive values of ΔH’ revealed that the adsorption process was endothermic [68].

## 4. Conclusion

Electrospun PAN nanofibers-based nanocomposites incorporated with fly ash and bentonite were developed at optimized process parameters to produce fine beadles’ nanocomposites. The optimization of process parameters was based on the nanofiber’s diameter. The optimized diameter of 110 nm was achieved at 30 kV of potential difference and 200 mm wire to collector distance. This optimization ensures the maximum exposure of nanocomposite with the Pb (II) ions. The batch adsorption studies showed that the removal of Pb (II) can be considerably affected by different factors. s

Moreover, the nanocomposite having the highest amount of fly ash exhibited the highest Pb(II) removal capacity compared with its counterparts. The equilibrium adsorption data was well explained by the pseudo-second order kinetic model, and the mechanism of adsorption was defined by Bangham’s model and the intra-particle diffusion model. The thermodynamic parameters indicated that the adsorption was endothermic and spontaneous. Based on the current study, the developed adsorbent can effectively treat domestic and industrial wastewater for Pb(II) adsorption. However, further studies are required to check the efficiency against other heavy metl.

## Figures and Tables

**Table 1:** Diameter of adsorbents.

Adsorbents	Average Diameter (nm)
PNF	113
NC1	116
NC2	110
NC3	112
NC4	119

**Table 2:** Composition of elements in PNF and nanocomposite (NC) samples.

Adsorbents	Element	Weight %
PNF	Carbon (C)	91.19%
	Oxygen (O)	4.78%
	Gold (Au)	4.03%
NC1	Carbon (C)	54.79%
	Oxygen (O)	28.08%
	Silicone (Si)	7.84%
	Aluminum (Al)	4.26%
	Calcium (Ca)	1.3%
	Gold (Au)	3.72%
NC2	Carbon (C)	57.6%
	Oxygen (O)	21.44%
	Silicone (Si)	9.41%
	Aluminum (Al)	5.46%
	Calcium (Ca)	1.05%
	Iron (Fe)	1.33%
	Gold (Au)	3.79%
NC3	Carbon (C)	58.09%
	Oxygen (O)	17.43%
	Silicone (Si)	10.54%
	Aluminum (Al)	7.75%
	Calcium (Ca)	1%
	Iron (Fe)	1.37%
	Gold (Au)	3.81%
NC4	Carbon (C)	59.74%
	Oxygen (O)	15.04%
	Silicone (Si)	10.99%
	Aluminum (Al)	7.99%
	Calcium (Ca)	0.98%
	Iron (Fe)	1.41%
	Gold (Au)	3.85%

**Table 3:** Kinetic modelling parameters of Pb (II) adsorption on electrospun PAN and cross-linked composites.

Kinetic Models	Parameters	Adsorbents				
		PNF	NC1	NC2	NC3	NC4
Pseudo-first order	q_e_	0.10	1.99	1.37	2.90	4.58
	K	0.005	0.009	0.007	0.01	0.02
	R^2^	0.82	0.86	0.87	0.89	0.89
Pseudo-second order	q_e_	0.315	4.59	3.09	5.99	8.17
	K	0.00045	0.47	0.105	0.03	0.015
	R^2^	0.99	0.99	0.99	0.99	0.99
Bangham’s	K	0.40	1.12	0.98	1.13	1.24
	β	14.25	10.36	11.59	8.76	8.22
	R^2^	0.71	0.80	0.91	0.89	0.83
Intra-particle diffusion	B_1_	0.08	0.77	0.91	1.69	3.21
	K_1_	0.01	0.17	0.08	0.104	0.08
	R^2^	0.93	0.97	0.99	0.96	0.73
	B_2_	0.25	3.63	2.39	5.18	7.51
	K_2_	0.0003	0.0042	0.0031	0.003	0.0012

**Table 4:** Thermodynamics parameters.

Adsorbents	ΔG’ (kJ·mol^−1^)						ΔS’ (J·mol^−1^·K^−1^)	ΔH’ (kJ·mol^−1^)
	293 K	298 K	303 K	308 K	313 K	318 K		
**PNF**	0.69	1.36	1.95	2.36	2.68	2.97	26.29	0.09
**NC 1**	−8.93	−9.27	−9.58	−9.87	−10.16	−10.37	7.88	0.06
**NC 2**	−8.26	−8.60	−8.87	−9.12	−9.36	−9.58	6.99	0.05
**NC 3**	−9.59	−9.92	−10.23	−10.51	−10.77	−11.04	7.28	0.06
**NC 4**	−10.44	−10.73	−11.01	−11.27	−11.52	−11.76	5.06	0.05
